# Interlaminar Microstructure and Mechanical Properties of Narrow Gap Laser Welding of 40-mm-Thick Ti-6Al-4V Alloy

**DOI:** 10.3390/ma15217742

**Published:** 2022-11-03

**Authors:** Xing Liu, Wanli Ling, Yue Li, Jianfeng Wang, Xiaohong Zhan

**Affiliations:** College of Materials Science and Technology, Nanjing University of Aeronautics and Astronautics, Nanjing 211106, China

**Keywords:** Ti-6Al-4V titanium alloy, narrow gap laser welding, interlayer microstructure evolution, numerical simulation, mechanical properties

## Abstract

Narrow gap laser welding (NGLW) is a common solution for the welding of thick structures. NGLW was carried out on narrow-gap butt joints of 40 mm-thick Ti-6Al-4V alloy plates with a U-shaped groove. The distribution characteristics of the interlaminar microstructure in different height ranges of the joint were investigated, and the evolution behavior and formation mechanism of the interlaminar microstructure of the joint were also revealed. This showed that a large amount of short needle martensite nucleated and grew up near the fusion line and the upper boundary of the remelting zone. The “softening” phenomenon occurred in all welds except the cover layer weld. The microstructure evolution and defect migration, induced by multiple welding thermal cycles in the upper weld forming process, were the main reasons for the “softening” of the lower weld. The tensile strength of each sample changed in the range of 920~990 MPa; the fracture mode of the sample belongs to a transgranular ductile fracture. In addition, compared with the upper part of the joint, the plasticity and toughness of the weld area in the lower part of the joint was improved.

## 1. Introduction

Titanium and its alloy, due to advantages such as high specific strength, high corrosion resistance, low density, high toughness and fatigue resistance, have been widely used in the field of aviation manufacturing [1,2,3]. The most widely used Ti-6Al-4V alloy is an ideal material for manufacturing titanium alloy tubing; it is especially suitable for aviation hydraulic systems and the petroleum industry [4,5]. Frame or beam components with a large size, unequal thickness, and a variable cross section are important parts of fuselage structures of aerospace vehicles [6,7]. Due to their large size, these structural components cannot be forged as a whole, so welding technology is particularly important. However, previous studies indicate that the existing manufacturing process of thick Ti-6Al-4V alloy parts has exposed several technical limits, such as the suppression of welding defects and the deformation controlling in the forming process [8,9,10].

Currently, the traditional arc-welding method still holds considerable importance due to its advantages of cost control and convenient operation [11]. However, there are many weaknesses in arc-welding, such as excessive deformation and tungsten inclusion [12]. Normally, the joint obtained by electron beam welding (EBW) is accompanied by a deeper penetration and a narrow heat-affected zone (HAZ). Nevertheless, the application of EBW is significantly limited by its vacuum condition [13,14]. Narrow gap laser welding applies a laser to melt a filler material and fill a narrow U-shaped groove between two plates, which is a highly efficient process especially for welding thick metal plates [15,16]. Many researches have reported that NGLW has superior ascendancy over other processes related to the welding of thick plates. According to Yu et al. [17], the technology of NGLW can overcome the penetration limit of autogenous laser welding by a layer-by-layer application of filler wire. Complementary studies by Sheriff et al. [18] demonstrated that the addition of filler material can compensate for the evaporation and burning-loss of elements during welding. Long et al. [19] put forward the principle of avoiding the poor fusion of the sidewall and the interlayer in narrow gap laser welding by using a filler of TC4 titanium alloy with a large thickness. As described in the study of Yang et al. [20], a defect-free welded joint of 100 mm-thick SUS 304 steel plates is fabricated by NGLW with filler wire in the laser conduction mode.

As a result of the multiple welding processes, NGLW is accompanied by complex thermal effects. In addition to the remelting of the lower weld bead, the intense thermal effect could provide a driving force for the phase transition in the vicinity of the fusion zone during the formation of the upper weld bead [21,22]. Jiang et al. [23] discussed the mechanism in pore formation and suppression by the interaction between the laser oscillation induced stirring effect and the solidification within the molten pool. According to the fundamental research on the Ti-6Al-4V alloy joint from Zhang et al. [24], the proportion of the primary α phase decreases and the proportion of the transformed β phase increases with the variation of temperature gradient from the near-base metal (BM) region to the near-HAZ region. The study of Haghdadi et al. [25] has revealed the effect of thermal variation on the crystallographic characteristics of α-α intervariant boundaries in the Ti-6Al-4V alloy parts fabricated by additive manufacturing. Furthermore, the sophisticated microstructure evolution will have implications for the service performance of the thick joint [26,27]. The fatigue crack growth rates of the laser powder bed fusion produced Ti-6Al-4V, which has been investigated by Becker et al. [28], and it is reported that the anisotropy of the fatigue properties is linked to morphological texture. Moreover, Maawad et al. [29] highlighted that the tensile performance differs in various welded sheets that have different volume fractions of grains with a certain orientation with respect to the welding direction. In conclusion, there are few NGLW studies on the microstructure evolution of the interlayer in different regions of the thick joint in the welding process.

In the current investigation, 40 mm-thick Ti-6Al-4V alloy plates were welded successfully via the NGLW by fiber laser. The microstructure evolution of the interlayer in different regions of the thick joint was identified. The relationship between the microstructure and the mechanical properties of the joint was discussed in detail.

## 2. Experimental Details

### 2.1. Materials

**Figure 1:**
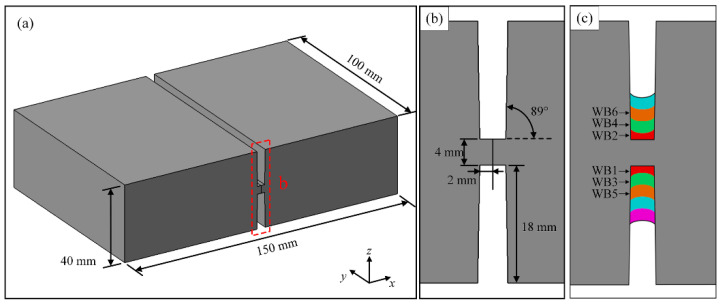
The schematic diagram of weldment: (**a**,**b**) the geometric dimension of 40 mm-thick Ti-6Al-4V alloy plates, (**c**) the welding sequence.

The material of base metal and welding wire used in the narrow gap laser welding experiment was Ti-6Al-4V alloy, the chemical composition and basic mechanical properties of which were obtained by the previous experiments listed in Table 1 and Table 2, respectively. The 40 mm-thick Ti-6Al-4V alloy plates were machined with a double-sided U-groove, and its specific geometric dimension is presented in Figure 1a,b.

### 2.2. Experimental Setup

**Figure 2:**
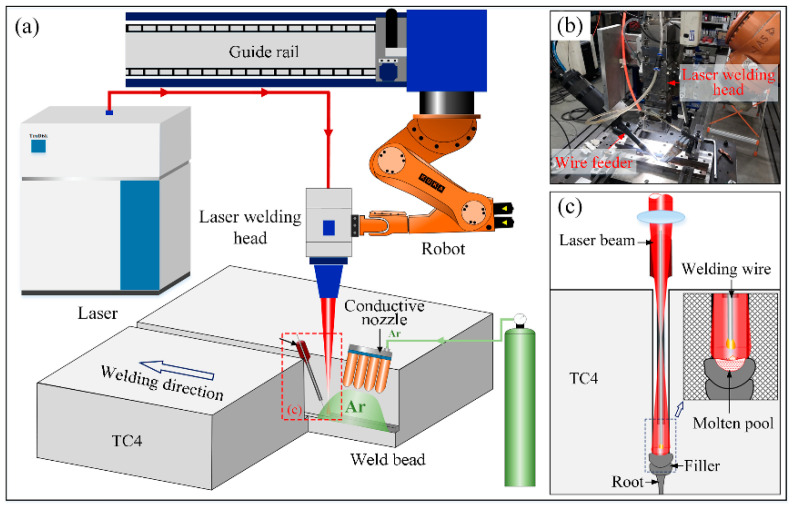
(**a**,**c**): the schematic diagram of the NGLW process; (**b**) the NGLW experimental equipment.

In the current work, the narrow gap laser welding experiment is performed by a KUKA HR60HA robot and a TruDisk-12003 Laser with the maximum output power of 12,000 W, produced by TRUMPF. The schematic diagram of NGLW is shown in Figure 2. A specially designed wire feed nozzle with a diameter of 3 mm was used, and the molten pool was protected by high purity argon during the laser welding process.

The whole welding process consisted of the formation of 21 layers of the weld bead. As presented in Figure 1c, the scanning sequence of different weld beads inside the symmetrical U-shaped groove was alternating. In order to guarantee the satisfactory formation of the weld bead and sidewall fusion, a high defocusing setting was experimentally considered. The specific welding parameters of the NGLW process are shown in Table 3.

### 2.3. Analysis Method

Following the welding process, the metallographic sample was cut along the cross-section of thick Ti-6Al-4V alloy plates, and an uneven area of the weld bead was avoided during the sample preparation. The 3D scanner (Prince-AXE, Scantech company) manufactured by Scantech was used to measure the deformation of the laser welded joint. The macroscopic and microscopic views in welded joints are, respectively, observed by a MR-5000 optical microscope (OM) after grinding the joints with abrasive paper, polishing them with diamond abrasion paste and etching them with Kroll reagent (2% HF + 8% HNO_3_ + 90% H_2_O).

**Figure 3:**
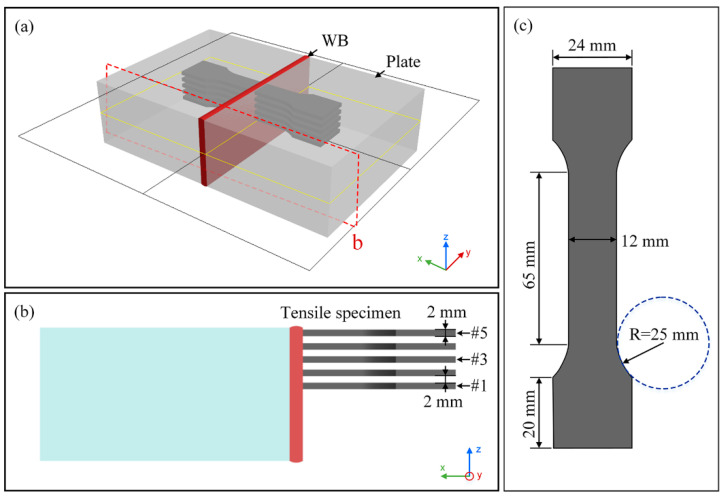
Tensile test specimens: (**a**,**b**) sampling location; (**c**) sample dimensions.

The Vickers microhardness measurements of different interlayers were conducted on a transverse cross-section of the 40 mm-thick Ti-6Al-4V alloy joint with a load of 200 g and a residence time of 15 s. Static tensile testing is an intuitive approach to reveal the mechanical behavior and basic performance indexes of metal materials such as yield strength, tensile strength, and elongation at break when subjected to static load [30,31]. The effect of the interlaminar microstructure on the mechanical properties and the fracture mechanism of the 40 mm-thick Ti-6Al-4V alloy joint was investigated. The tensile experiments are performed with a displacement rate of 0.8 mm/min at an ambient temperature (25 °C) using a universal testing machine (UTM). After the tensile experiment, scanning electron microscopy (SEM) is adopted to observe the fracture morphology, while energy dispersive spectroscopy (EDS) is employed to characterize the element. Figure 3 marks the geometric dimension of the test specimen and the sampling location.

## 3. Result and Discussion

### 3.1. Weld Formation

**Figure 4:**
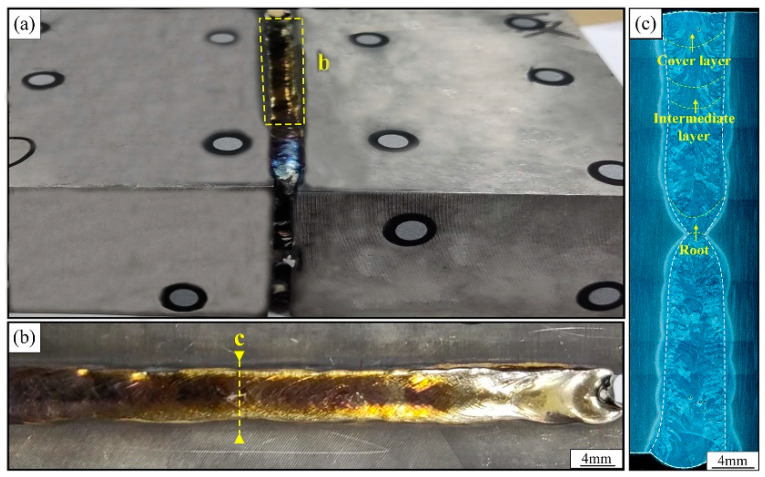
Macro forming of the 40 mm-thick TC4 titanium alloy narrow gap laser welded joint: (**a**) the macro forming of welded joint, (**b**) the cover layer, (**c**) the joint cross-section.

Figure 4 shows the macroscopic shapes of the weld seam and the joint cross-section; the cover layer weld is oxidized slightly, the weld surface is faint yellow. The filler material was well-spread in the groove and fully fused with the base material from both sides. There are only a few porosity defects in the joint cross-section, and the diameter of the porosity does not exceed 0.2 mm. The fusion between the layers and the side walls of the joint is adequate, and the fusion width of the weld is within the range of 7–7.5 mm. The height of the cover layer is about 3.5 mm and the height of other filling welds is about 1.8–2.0 mm.

**Figure 5:**
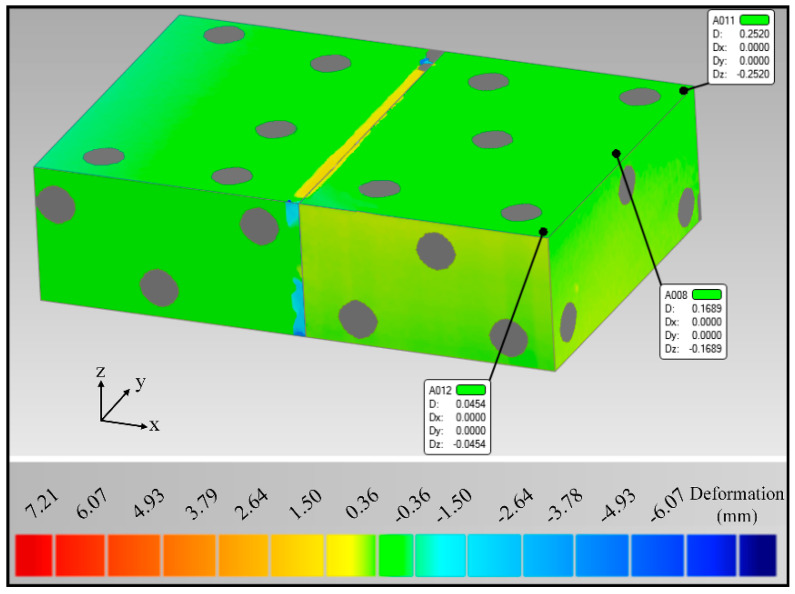
Deformation cloud after welding of the 40 mm-thick TC4 titanium alloy narrow gap laser welded joint.

Comparing the original model with the deformation data, the joint deformation cloud is depicted in Figure 5, disregarding the displacement deviation caused by the unfilled position of the bevel, and extracting the deformation data only for the edge of the test plate. The deformation results show that there is a slight warpage of the test plate after welding. The maximum deformation of the test plate edge is only 0.25 mm, and the maximum warpage angle is calculated to be about 0.19°. Therefore, the symmetrical U-shaped groove was used, and the alternating welding sequence can suppress effectively the deformation after welding.

### 3.2. Microstructure Examination

**Figure 6:**
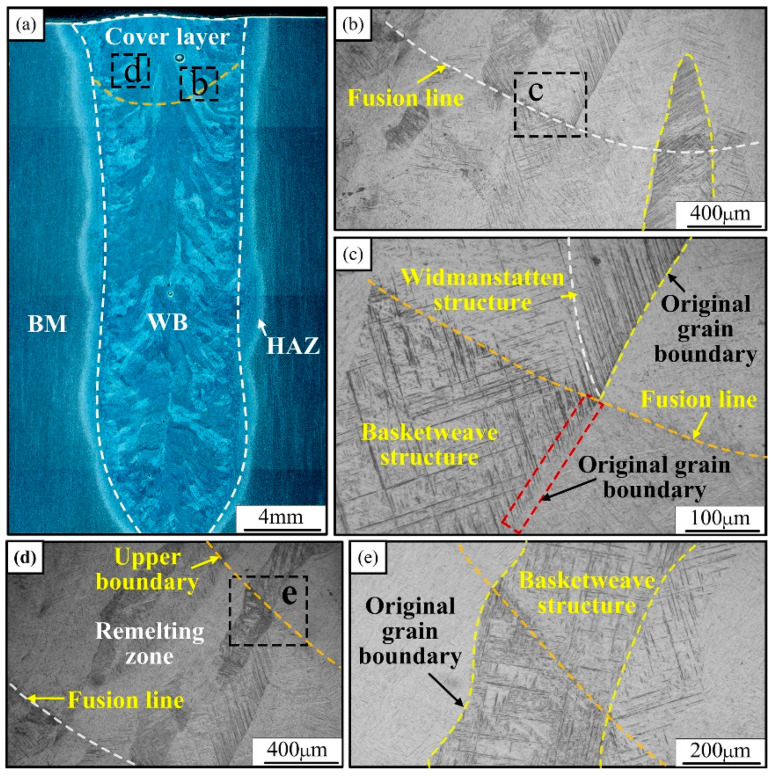
Microstructure of the upper interlayer weld area of the joint: (**a**) the joint cross-section, (**b**,**c**) the microstructure near the fusion line, (**d**,**e**) the microstructure near the upper boundary of the remelting zone.

The microstructure of the interlaminar weld zone is intercepted and studied in three different areas of the upper, middle, and lower parts of the joint, respectively. The interlayer microstructure characteristics of different areas of the joint are analyzed, and the variability of the microstructure of the weld zone, between different areas of the layers, is quantitatively compared. Figure 6 demonstrates the interlayer microstructure of the cover layer. As seen in Figure 6d,e, there is a distinct upper boundary of the remelting zone and of the fusion line in the upper interlayer region of the joint. The measured statistics show that the width of the remelting zone is in the range of 900~1000 μm. Figure 6b,c shows the microstructure near the interlayer fusion line, and it can be seen that a short needle-like α’ phase appears near the fusion line. Above it (i.e., the part of the cover layer weld), the widmanstatten structure consisting of long and wide clusters of needle-like martensite is regularly arranged: the white color is the needle-like α’ phase and the black border between the phases is the β phase. Below the fusion line, the basketweave structure consisting of the cross-arrangement of needle-like martensite appears inside the coarse β columnar crystal. In addition, the second welding heat cycle in the upper weld, the fusion line below the part of the original β columnar grain boundary, gradually tends to be “blurred”. Figure 6d,e show the microstructure near the upper boundary of the remelting zone. As can be seen from the figure, the boundary traverses the growing β columnar crystal, but the upper and lower sides of the boundary are netted tissues composed of acicular martensite crossing, and there is no tissue difference below the fusion line.

**Figure 7:**
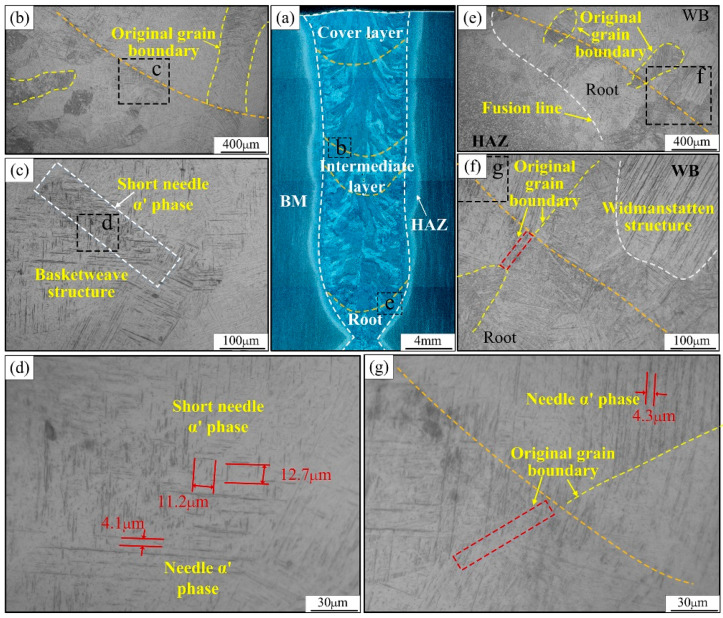
Microstructure of the middle and the lower interlayer weld area of the joint: (**a**) the macromorphology, (**b**,**c**) the interlayer microstructure in the middle of joint, (**d**,**g**) the needle-like α’ phase morphology, (**e**,**f**) the interlayer microstructure near the root of joint.

Figure 7 shows the interlayer microstructure morphology of the middle and lower part of the joint. Unlike the interlayer organization of the upper part of the joint, the upper boundary of the remelting zone in the middle and lower part disappears completely due to the occurrence of multiple solid-state phase transformations. Therefore, in this part, the interlayer microstructure near the fusion line of the two regions was intercepted for analysis. Figure 7c,d show that short needle-like martensite tissue also appears near the fusion line in the middle of the joint. As shown in Figure 7d, the martensite length dimensions of ten groups in the field of view are randomly performed, and the average martensite length near the fusion line is around 12 μm. Figure 7f,g show that although the width dimension of the columnar crystals in the lower part of the joint is smaller, the widmanstatten structure, consisting of coarse needle-like martensite clusters, still appears above the fusion line. In addition, as seen in Figure 7g, the original β columnar grain boundary below the fusion line in the lower part of the joint has nearly disappeared due to the occurrence of multiple solid-state phase transformations. There are some differences in the growth trend behavior and size of β-columnar crystals in different regions of the joint. It was found that the growth trend of β columnar crystals in the weld zone at the lower part of the joint points to the weld center and it is approximately parallel to the horizontal plane. The β-columnar crystals in the weld zone in the middle and upper part of the joint show two growth trends, vertical upward and inclined upward, and the β-columnar crystals growing in the inclined upward trend also show obvious growth corners.

**Figure 8:**
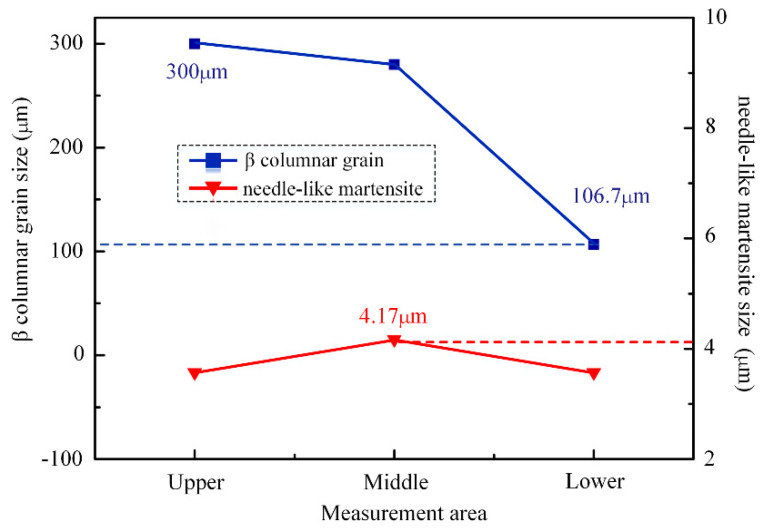
Results of quantitative analysis on the microstructure of the weld zone between layers in different areas of the joint.

To quantitatively analyze the difference of the microstructure characteristics between layers in different regions of joints, the intercept method was used. This measured the average width size of acicular martensite in the Weihtensite structure of the head, middle and lower regions. After 5 groups of data were measured, the average width of spiculate martensite was calculated. The measurement results of needle martensite width are shown in Figure 8. The results show that the width of needle martensite in different regions is about 4.0 μm, and the size difference is not large, and does not show obvious change law. The size of the β columnar crystal in the lower part of the joint is obviously lower than that in the upper and middle part of the joint. The average width of the β columnar crystal in the weld area of the lower part of the joint is 106 μm, which is only one third of the width of the β columnar crystal in the middle and upper part of the joint.

**Figure 9:**
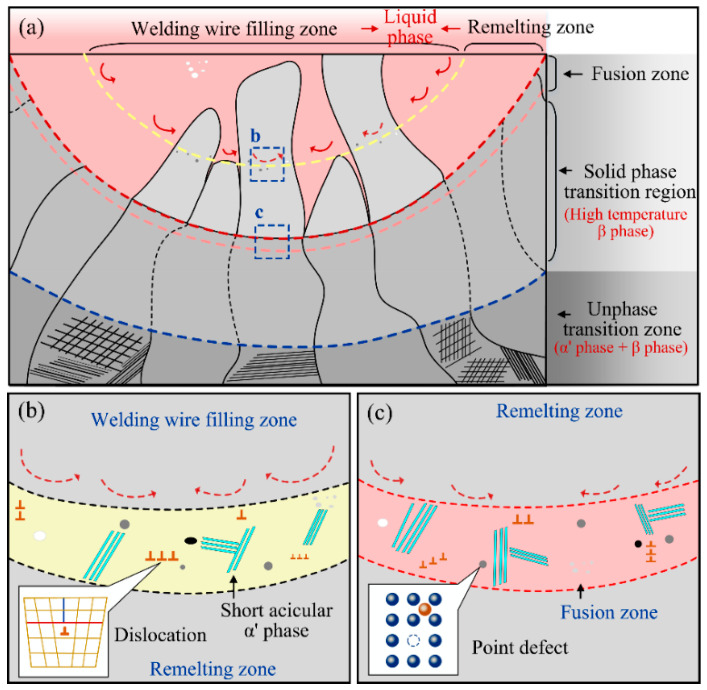
Analysis of the mechanism of the microstructure evolution in the interlayer weld area: (**a**) diagram of the weld zone, (**b**) microstructure evolution near the upper boundary of remelting zone, (**c**) microstructure evolution of the fusion zone.

The formation mechanism of the short needle-like α’ phase near the upper boundary of the remelting zone and the fusion line was studied, as shown in Figure 9. The Ti-6Al-4V titanium alloy laser welding is a rapid heating and cooling process. In the process of rapid cooling of the high temperature β phase, a solid solution forms, in which the alloying elements are too late to diffuse. At this point, the occurrence of the martensitic phase transformation belongs to the non-diffusion type phase transformation. Many studies have shown that, similar to the liquid-solid phase transformation, the martensite phase transformation is also a nucleation and growth process [8]. Therefore, this paper analyzed the formation mechanism of the short needle-like α’ phase from the perspective of nucleation.

As shown in Figure 9a, during the narrow gap laser welding of the Ti-6Al-4V titanium alloy, the liquid molten pool can be divided into two parts: the lower weld, which is secondarily melted by the laser heat, and the new weld, which is formed by filling in the molten wire. During the formation of the upper weld, the surface area of the lower weld absorbs the laser energy and remelts. Subsequently, the wire melts and transitions into the molten pool under the action of the laser beam. During the transition of the molten droplet, a strong stirring effect on the formed molten pool is inevitable, as shown in Figure 9b. Therefore, near the upper boundary of the remelting zone, there is obvious energy, structural as well as compositional undulations, which provide the nucleation conditions for the subsequent martensite phase transformation. At the same time, after solidification of the weld, a large number of defects such as interstitial atoms, dislocations, and laminations are retained near the boundary of the remelting zone due to the stirring behavior of the melt pool. When the high temperature β phase cooled to the phase transition temperature, the high-density defects will provide many nucleation conditions for the formation of the α’ phase. Near the boundary, the α’ phase first nucleates and grows at the location of the point defects, line defects, etc. As a result, a large number of short needle-like α’ phases are formed near the boundary of the remelting zone.

The formation mechanism of short needle-like α’ phases near the fusion line is similar to the above-mentioned case. As shown in Figure 9c, compared with the upper boundary of the remelting zone, the stirring behavior of the molten pool near the fusion line is relatively weakened. However, after liquid solidification, the original solid-liquid interface also retains a certain number of nucleation prone positions such as point defects, dislocations and stacking faults. In the meantime, because it is close to the base metal, the undercooling near the fusion line is higher. Many studies show that when the undercooling increases, the critical nucleation decreases successfully, which means that the occurrence of nucleation increases. Therefore, a large number of α′. The phase nucleates and grows near the original solid-liquid interface, forming a short needle shape α′. The fusion zone formed by aggregation is shown in Figure 9c.

**Figure 10:**
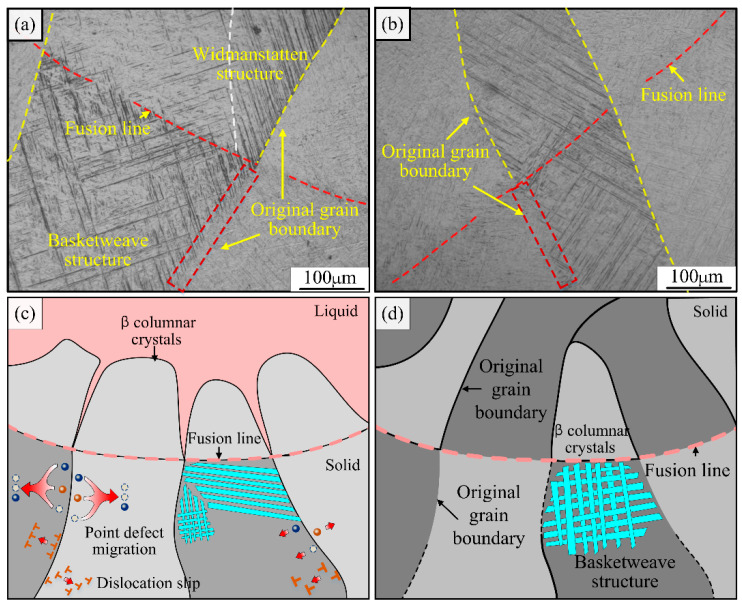
Analysis of the mechanism of the influence of the welding thermal cycle characteristics on the heat-affected zone below the fusion line: (**a**) cover layer, (**b**) intermediate layer, (**c**) microstructure evolution of the original β grain boundary during welding, (**d**) microstructure of the original β grain boundary after welding.

Meanwhile, some of the β columnar crystals below the fusion line showed fuzzy or even disappearing grain boundaries, and this phenomenon appeared in several interlayer weld regions such as the upper, middle, and lower parts of the joint, as shown in Figure 10a,b. To explain the above phenomenon, this study was carried out to investigate the microstructure evolution behavior of the heat-affected zone below the fusion line. As seen in Figure 10a,b, the grain boundary disappearance region is located within 200 μm below the fusion line, and this region is subject to the same strong thermal action during the forming of the upper layer weld, although remelting does not occur. Due to the different cooling conditions, the microstructure before and after the solid-state phase transformation may differ; at this moment the original β columnar grain boundary morphology is weakened.

In addition, the grain boundary is a collection of many point defects and line defects (dislocations). First, the local area below the fusion line experiences a sudden temperature rise and increased atomic activity due to the thermal influence of the upper weld. As a result, point defects concentrated at grain boundaries start to migrate under the action of a driving force, and some vacancy defects migrate to dislocations, or disappear in migration by combining with interstitial atoms. At the same time, due to the increased atomic activity in addition to point defects, dislocations in the role of internal stresses in the weld are activated and slip, while the slip process of dissimilar dislocations occurs in combination and offset. Therefore, the grain boundary near the point defects, and line defects’ density will be further reduced. In summary, due to the tissue evolution of the secondary solid state phase transformation and the influence of defect migration, the fusion line below part of the original β columnar grain boundaries, in the upper layer of the weld forming process under the influence of heat, gradually tends to blur or even disappear.

### 3.3. The Weld Joint Mechanical Properties

**Figure 11:**
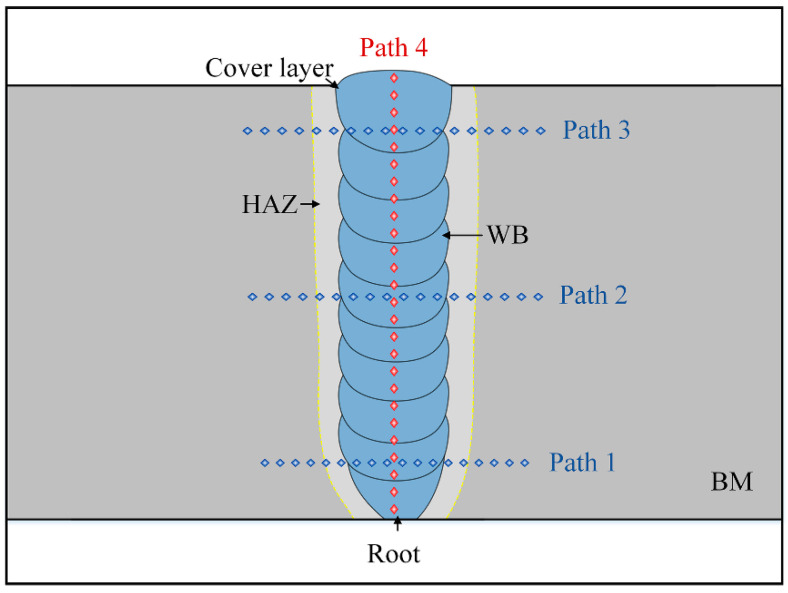
The Vickers hardness test path selection diagram.

**Figure 12:**
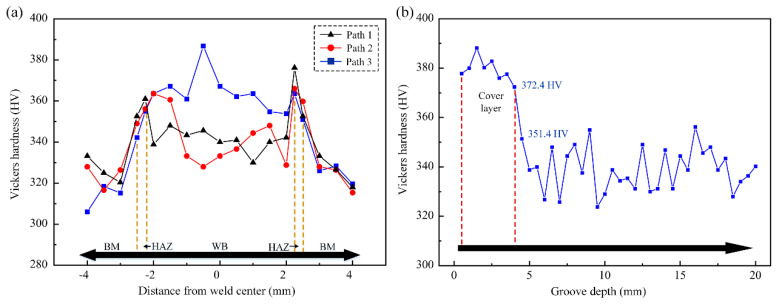
The Vickers hardness test results of welded joints: (**a**) path 1—path 3, (**b**) path 4.

Figure 11 shows the path selection schematic for the micro-Vickers hardness test, and a total of four paths were selected. The hardness test results are shown in Figure 12. The results show that the joint of the heat-affected zone and weld hardness are much higher than the base material after the martensitic phase transformation is completed. The weld area below the cover layer shows an obvious “softening” phenomenon. The average hardness of the cover layer weld is 379.4 HV, while the average hardness level of the other layers of the weld is only 339.1 HV. Analysis shows that the upper layer of the weld forming process of multiple welding thermal cycle-induced tissue evolution and defect migration behavior is the main reason for the “softening” of the lower layer of the weld.

**Figure 13:**
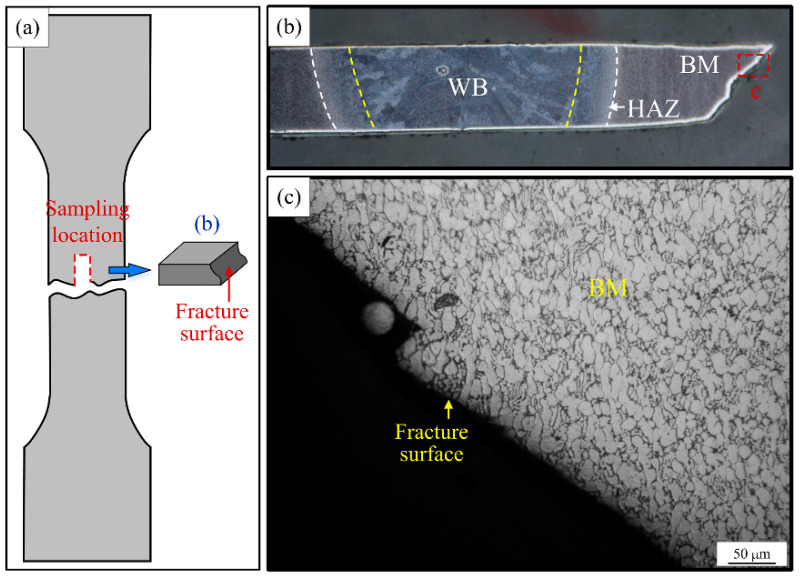
Macroscopic morphology and microstructure of the tensile specimen cross-section at failure position: (**a**) the schematic diagram of metallographic sampling location, (**b**) the macroscopic appearance of the cross section of the failure location, (**c**) the microstructure morphology.

In order to further investigate the influence of interlayer tissue variability in different areas of the joint on the mechanical properties, the microstructure of specimen #5 was analyzed. The failure of the specimens occurred in the parent material area, and the microscopic morphology near the fracture surface of specimen #5 is shown in Figure 13. The fracture surface penetrates many equiaxed grains in the base material area. Therefore, the fracture mode of the tensile specimens can be tentatively judged as a grain penetration ductile fracture.

**Figure 14:**
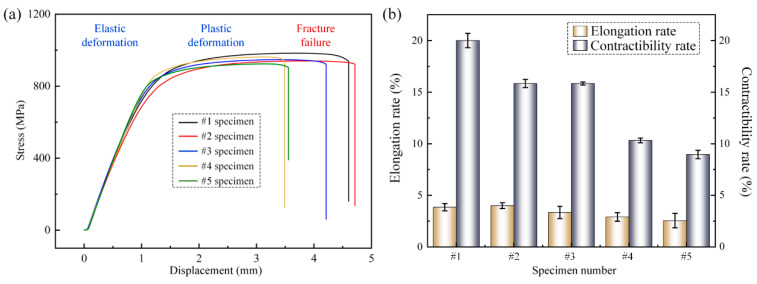
Tensile property test results: (**a**) the displacement-stress variation curve of tensile specimens, (**b**) the elongation at break and the shrinkage at section of tensile specimens.

Figure 14a shows the displacement-stress variation curves of different tensile specimens. During the tensile process, with displacement increased, the specimen first enters the elastic deformation stage in which the stress level increases linearly. Subsequently, when the specimen deformation exceeds the critical value, the tensile specimen enters the plastic deformation stage in which the deformation is irreversible. Finally, with the increasing loading force, the microcracks inside the specimen continued to expand to the critical length and the specimen failed to fracture. Comparing the test results of tensile specimens sampled from different areas of the joint, the tensile strength of the specimens ranged from 920 to 990 MPa, and the tensile strength of different specimens did not vary much and did not show an obvious pattern of change.

The post-break length L and post-break cross-sectional area A were measured for the failed specimens, and the results are shown in Figure 14b. The measurement results showed that specimen #1 had the highest shrinkage at break, reaching 20%, while specimen #5, which was sampled from the upper part of the joint, had a shrinkage at break of only 8.96% as the sampling position increased. Similarly, the elongation after break showed the same trend, with specimens #1 and #2, which were sampled from the lower part of the joint, showing an elongation after break of about 4%, while the elongation after break of specimen #5 decreased to 2.55%. Tensile specimens can be divided into three parts: base material, heat-affected zone and weld zone, and all specimens have the same mechanical properties base material, so different specimens in the tensile test show plasticity variability associated with the weld, heat-affected zone.

**Figure 15:**
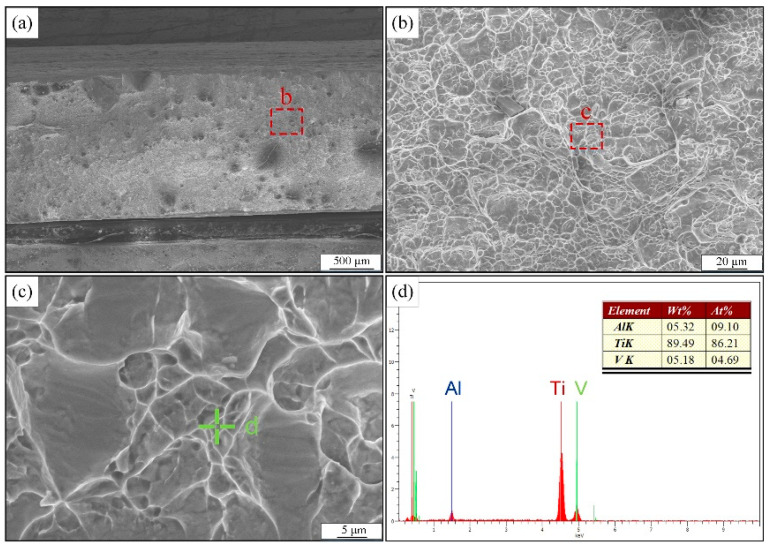
Fracture microscopic morphology and the composition of tensile specimens: (**a**–**c**) the SEM results, (**d**) the EDS result.

The fracture morphology observation of the tensile specimen was carried out, as shown in Figure 15. From the figure, the fractures are fibrous and show a grayish color. The fracture areas are all equiaxed tough nests, so one can judge that the fracture modes of the tensile specimens all belong to the microporous aggregation type of toughness fracture. In addition, the composition scan of the local area of the fracture shows that the mass fraction of the V element in the fracture section has increased, while the content of the Al element has decreased.

## 4. Conclusions

The microstructure evolution of the interlayer in different regions of the thick joint, and the relationship between the microstructure and the mechanical properties of the Ti-6Al-4V alloy manufactured by the narrow gap laser welding by fiber laser, have been investigated in detail. The following specific conclusions can be drawn.

A Ti-6Al-4V alloy with 40 mm-thick was successfully welded by NGLW. The welds were well formed and high-quality, defect-free joints were obtained. The results of the three-dimensional scanning deformation test of the test plate show that the edge of the test plate has warping deformation; the maximum deformation is only 0.25 mm, and the deformation angle is 0.19°. It shows that the double-sided U-groove and alternate welding order can effectively restrain the deformation of the plate after welding.

There is still a clear upper boundary of the remelting zone at the lower part of the cover layer weld. Except for the cover layer weld, the upper boundary of the remelting zone between other weld layers disappears completely after multiple welding thermal cycles. Many short needles are formed near the boundary of the remelting zone and fusion line α′ phase.

The hardness test results show that there is an obvious “softening” phenomenon in the weld area below the cover layer. The microstructure evolution and defect migration, induced by multiple welding thermal cycles in the upper weld forming process, are the main reasons for the “softening” of the lower weld.

The tensile property test results show that the tensile strength of each sample changes in the range of 920~990 MPa. The fracture surface and characteristics show that the fracture mode of the sample belongs to a transgranular ductile fracture. In addition, the plastic toughness of the weld zone in the lower part of the joint is improved significantly, compared with that in the upper part.

## Figures and Tables

**Table 1:** Chemical composition of the Ti-6Al-4V alloy.

Composition	Fe	C	H	O	N	Al	V	Ti
wt.%	≤0.30	≤0.10	≤0.015	≤0.20	≤0.05	5.5–6.8	3.5–4.5	Balance

**Table 2:** Mechanical properties of the Ti-6Al-4V alloy.

Material	Tensile Strength (MPa)	Yield Strength (MPa)	Elongation (%)
Ti6Al4V alloy	980	938	14.6

**Table 3:** The welding parameters of the NGLW process.

Process Parameters	Amount
Laser power (kW)	4.5
Welding speed (m/min)	0.9
Wire feeding speed (m/min)	9
Defocusing (mm)	15

## Data Availability

Data and materials are available.
